# Endovascular Aneurysm Repair in HIV Patients with Ruptured Abdominal Aneurysm and Low CD4

**DOI:** 10.1155/2016/8572950

**Published:** 2016-09-15

**Authors:** Saranat Orrapin, Saritphat Orrapin, Supapong Arworn, Termpong Reanpang, Kittipan Rerkasem

**Affiliations:** ^1^Division of Vascular and Endovascular Surgery, Department of Surgery, Faculty of Medicine, Chiang Mai University, Chiang Mai, Thailand; ^2^NCD Center, Faculty of Medicine, Chiang Mai University, Chiang Mai, Thailand; ^3^NCD Center of Excellence, Research Institute of Health Sciences, Chiang Mai University, Chiang Mai, Thailand

## Abstract

We report two HIV infected patients with ruptured abdominal aneurysm by using endovascular aneurysm repair (EVAR) technique. A 59-year-old Thai man had a ruptured abdominal aortic aneurysm and a 57-year-old man had a ruptured iliac artery aneurysm. Both patients had a CD4 level below 200 *μ*/L indicating a low immune status at admission. They were treated by EVAR. Neither patient had any complications in 3 months postoperatively. EVAR may have a role in HIV patients with ruptured abdominal aneurysm together with very low immunity.

## 1. Introduction

Improvement and early initiation of antiretroviral (ARV) drug had reduced deaths from human immunodeficiency virus (HIV) [1, 2]. However, HIV infection can still lead to many complications which cause death such as aneurysm formation [3]. Ruptured large vessel aneurysm formation such as infrarenal abdominal aortic aneurysm (AAA) and iliac aneurysms has associated with high mortality [4].

The best treatment for HIV infected patients with ruptured abdominal aneurysm with low white blood cell clusters of differentiation 4 (CD4) level is still controversial. Using data from the last 20 years, literature reports that patients without evidence of acquired immunodeficiency syndrome (AIDS) should be managed as standard vascular practice. For full-blown HIV patients, prone to AIDS or low immunity (CD4 < 200 *μ*/L) and with severe comorbidity, conservative therapy was suggested due to the high morbidity and mortality associated with open surgery [3, 4]. However, the survival analysis from our recent series of case studies of HIV infection associated vasculopathies showed a high mortality rate of large vessel aneurysm patients with nonoperative management [5].

At present, endovascular aneurysm repair (EVAR) results in lower perioperative morbidity and mortality compared to conventional open surgery for treatment of ruptured abdominal aortic and iliac aneurysms [6]. Recent literature shows that HIV infected patients with a CD4 = 130 *μ*/L could be treatment by EVAR [7]. Due to the fact that only a few case reports show successful EVAR of ruptured infrarenal AAA in HIV patients, our study aimed to describe EVAR in HIV infected patients with ruptured aneurysm.

## 2. Case Summary

HIV infected patients, who have had EVAR due to ruptured abdominal aneurysm, from 1 January 2009 to 31 October 2015, were included in the study. Clinical data, radiological findings, and perioperative complications and outcomes were extracted. Patients were followed up to 6 months after EVAR. This study was approved by our local ethics committee (study code: SUR-2558-03060). Two (2) cases of HIV infected patients who had had EVAR performed were included. The first case had a ruptured AAA and the second a left common iliac artery aneurysm. The details of the cases are shown in Table 1.

### 2.1. Case 1

**Figure 1:**
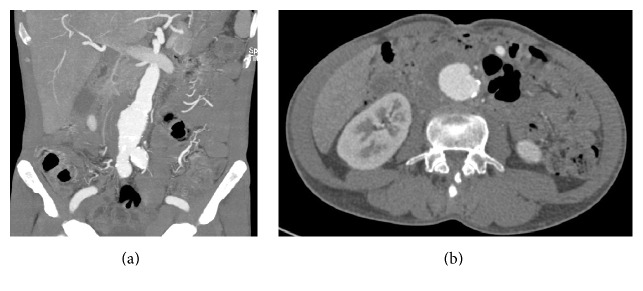
First patient: CTA abdomen. (a) Coronal section view of CTA: wall enhanced infrarenal AAA with periaortic tissue thickening and hematoma. (b) Cross-sectional view from CTA: blistering area of the aneurysm wall.

A 59-year-old Thai man visited the emergency department complaining of abdominal pain, fever for one day, and a weight loss of 5 kilograms (kgs) in one week. An expansile and pulsatile mass with marked tenderness was found in the mid part of his abdomen. His body temperature was 37.8°C. A white blood cell count (WBC) showed mild leukocytosis and the erythrocyte sedimentation rate was 112 millimetres/hour (mm/hr). His computerized tomographic angiography (CTA) showed the wall enhanced infrarenal AAA 4.8 × 4.0 cm with periaortic tissue thickening and a hematoma (Figure 1(a)) with small blistering of aneurysm (Figure 1(b)). The contained rupture of infected infrarenal AAA was diagnosed. An anti-HIV test was deemed positive using a reaction of enzyme-linked immunosorbent assay (ELISA), chemiluminescent microparticle immunoassay (CMIA), and HIV determination (3 methods). The CD4 level was 154 cells/mm^3^ (12%). Intravenous (IV) ciprofloxacin and clindamycin were started on the admission date for treatment of the infected AAA. His hemoculture was negative. The EVAR was performed as an emergency procedure by ENDURANT II® AAA bifurcated Stent Graft System (Medtronic, Inc.). Angiography carried out on completion showed no evidence of endoleak. The clinical fever was improved and the patient was discharged from hospital. There were no other perioperative complications from the discharge date until 6 months after treatment.

### 2.2. Case 2

A 57-year-old man presented with chronic febrile illness and weight loss for 2 months. He had developed a painful bulging mass in his left lower abdomen 8 cm in size. His body temperature was 39.0°C. He had a history of hypotension. CTA showed the contained ruptured left CIA aneurysm. His basic laboratory showed leukocytosis (white blood cell count of 18,600 cells/mm^3^) and acute renal failure (creatinine 7.5 milligrams/decilitre).

**Figure 2:**
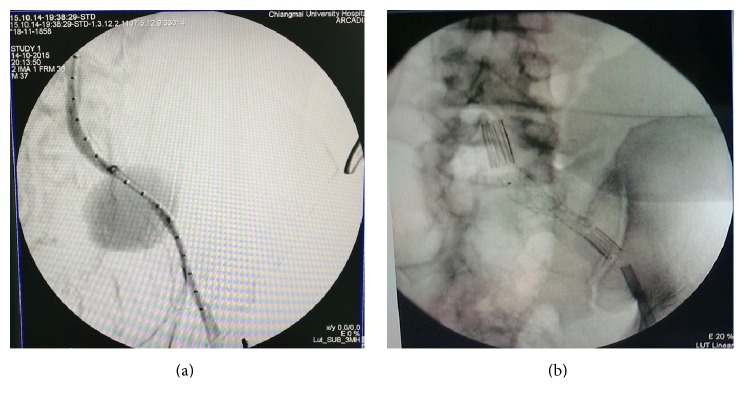
Intraoperative angiography. (a) showed angiogram with large concealed rupture in the left common iliac artery aneurysm in the second case. (b) showed angiogram after left iliac stent covered leakage area.

The anti-HIV test was positive. The CD4 level = 147 cells/mm^3^ (10%). He received emergency treatment by angiography and iliac stenting with ZENITH FLEX® endovascular graft iliac leg extension system (Cook Medical, Inc.). The EVAR was carried out without any endoleak or other complications (Figure 2). Although his hemoculture was negative, he was still given intravenous antibiotics for empirical treatment for 6 weeks. He was discharged from the hospital 6 weeks during intravenous antibiotic treatment and then he was switched to oral antibiotic therapy without other complications until 6 months after treatment.

## 3. Discussion

The most common cause of aortic and iliac aneurysm is atherosclerosis and degenerative changes which usually occur in old aged people. Our study showed atypical presentations of aneurysms in HIV infected patients including the onset of aneurysm in the young. Large vessel vasculopathy causes aneurysm in people of all ages. In general, major pathologic role of aneurysm formation is leukocytoclastic vasculitis of the vasa vasorum with chronic inflammation leading to fragmentation of internal elastic lamina. Secondary bacterial infection hypothesized is still controversial [8]. All HIV infected patients with ruptured large vessel aneurysms may lead to high morbidity and mortality with conventional open repair that may cause many physiological disturbances to the patients in particular when the immunity of patients is poor. Our study showed the good outcome without any complication of EVAR in ruptured large vessel aneurysm of low CD4 patients until 6 months after treatment.

In many reports, EVAR reduced morbidity and mortality of non-HIV related AAA repair in emergency cases but it is still in question regarding ruptured aneurysms in HIV infected patients. There are very few reports regarding treatment in HIV infected patients with large vessel aneurysm [7, 9, 10]. Only one case report described a ruptured large vessel (iliac artery) true aneurysm in HIV infected patient as discussed by Aziz et al. [10]. This study showed that it was safe to carry out EVAR for such patients without any complications. The 30-day perioperative outcomes were good. The CD4 level in the report by Aziz et al. is 581 cells/*μ*L, whereas our two cases had very low CD (CD4 < 150 cells/*μ*L). The results of the treatment were not affected by the patient's CD4 level. It was possible to reduce patient physiological disturbance by this less invasive procedure (EVAR). Also EVAR reduced the possibility of blood transmitted disease to the operating team.

Pathophysiological mechanisms of aneurysm formation in HIV patients have not been clearly determined with no evidence of degenerative disease from the aneurysm specimen. The association between AAA and HIV infection may be caused by bacterial infection resulting from immunosuppression or direct viral action. HIV patients are at risk of microbial infection which may lead to arteritis or secondary mycotic aneurysm formation after bacteria have lodged in the arterial wall [3, 4]. However, cultures grown from the aneurysm wall or thrombus yielded positive results in only 20% of cases [3].

The limitation of this study is a case series that included only two patients. Therefore, the further more sample size studies would be required and produce a more reliable outcome.

## 4. Conclusion

EVAR may be an alternative treatment for emergency HIV infection associated ruptured large vessel aneurysm. The 6-month postoperative result of treatment appeared to be equivalent to that of non-HIV patients and this result was not affected by the patient's CD4 level.

## Figures and Tables

**Table 1:** Summary of the baseline clinical data, management, and outcome.

Patient number	Age	Gender	CD4^+^ count^*∗*^ (cells/*µ*L)	Presentation	Vessel morphology	Comorbidities	H/C	Management	Outcome
1	59	Male	112	Fever + abdominal pain + pulsatile mass	Rupture of fusiform infrarenal AAA	None	No growth	(i) Intravenous ciprofloxacin + clindamycin × 6 weeks (ii) ENDURANT II AAA endovascular graft bifurcated system (Medtronic, Inc.)	(i) Improved without complications

2	57	Male	147	Fever + abdominal pain + hypotension	Rupture of fusiform left CIA aneurysm	(i) AKI with hyperkalemia	No growth	(i) Intravenous ciprofloxacin + clindamycin × 6 weeks (ii) ZENITH FLEX endovascular graft iliac leg extension system (Cook Medical, Inc.)	(i) Improved without complications

^*∗*^Both cases were low immunity HIV patients with CD4 < 200 cells/*µ*L; echocardiogram workup: no infective endocarditis or valvular heart disease and no incidence of intracardiac clots or thrombi.

CD4^+^: white blood cell clusters of differentiation 4 (CD4) level; *µ*L: microlitres; H/C: hemoculture; AAA: abdominal aortic aneurysm; CIA: common iliac artery; AKI: acute kidney injury.
